# Scapular Tuberculosis

**DOI:** 10.4269/ajtmh.25-0313

**Published:** 2025-08-26

**Authors:** Kenta Nakamoto, Shinnosuke Fukushima, Kohei Oguni, Hideharu Hagiya

**Affiliations:** Department of Infectious Diseases, Okayama University Hospital, Okayama, Japan

**Keywords:** *Mycobacterium tuberculosis*, Osteo-articular tuberculosis, cold abscess

**Figure 1.**
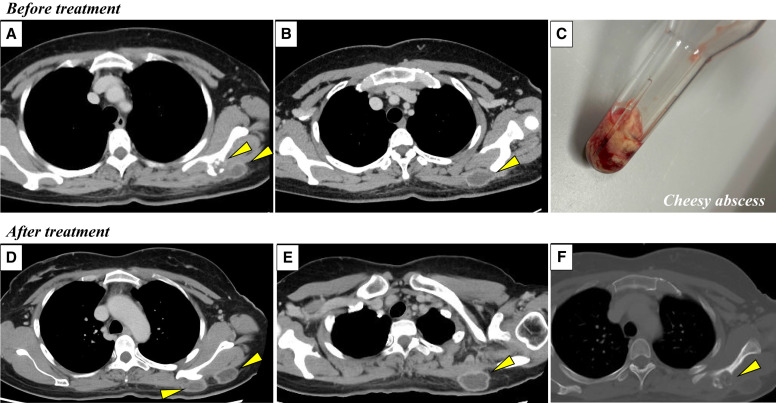
Radiologic and gross findings. (**A** and **B**) Axial CT image demonstrating multiple abscess lesions measuring 25 mm in diameter with associated osteolytic changes in the left scapula (arrowheads). (**C**) Gross specimen photograph showing purulent material with characteristic cheesy appearance consistent with caseous necrosis. (**D** and **E**) Follow-up contrast-enhanced CT image revealing progression of subcutaneous abscesses with increased size (arrowheads). (**F**) Follow-up contrast-enhanced CT image depicting progressive osseous destruction of the left scapula (arrowhead).

A 44-year-old Filipino woman without remarkable underlying disease presented with progressive left shoulder tenderness that developed over several weeks. Radiological investigations revealed multiple abscess lesions measuring 25 mm, accompanied by osteolytic changes in the left scapula (Figure 1A and B). Laboratory investigations demonstrated positive results for interferon-gamma release assay, and *Mycobacterium tuberculosis* (TB) was isolated from the bacterial culture of an aspirated cheesy abscess (Figure 1C). Under the diagnosis of osteoarticular tuberculosis, the patient underwent standard multi-drug antitubercular therapy (Rifampin [450 mg/day], Isoniazid [300 mg/day], Pyrazinamide [1,300 mg/day], and Ethambutol [750 mg/day]). Four weeks later, she experienced increased tenderness and restricted range of motion in her left shoulder region, with imaging revealing enlargement of the TB lesions (Figure 1D–F). Surgical intervention with local incision and drainage yielded caseous material, after which the patient demonstrated clinical improvement. Mycobacterial culture of the specimen was negative, suggesting a paradoxical inflammatory response rather than treatment failure of anti-TB therapy.

This case exemplifies the diverse manifestations of *M. tuberculosis*. Osteoarticular involvement occurs in approximately 2% of all TB patients, representing an uncommon but clinically significant extrapulmonary manifestation of the ubiquitous pathogen.^1^ Spinal involvement constitutes the predominant presentation of the osteoarticular TB infection, while shoulder joint infection is exceedingly rare,^2^^,^^3^ and reported in <2% of all osteoarticular TB cases.^1^ Patients with osteoarticular TB infection manifest only nonspecific clinical symptoms, and making a definite diagnosis is challenging.^4^
